# Preparation and Physicochemical Properties of a New Biolubricant from Epoxidized Fatty Acids and Diethylene Glycol Monomethyl Ether

**DOI:** 10.1021/acsomega.5c01223

**Published:** 2025-04-29

**Authors:** Kanokwan Chaiendoo, Jeerati Ob-Eye, Vorranutch Itthibenchapong

**Affiliations:** National Science and Technology Development Agency (NSTDA), National Nanotechnology Center (NANOTEC), Pathum Thani 12120, Thailand

## Abstract

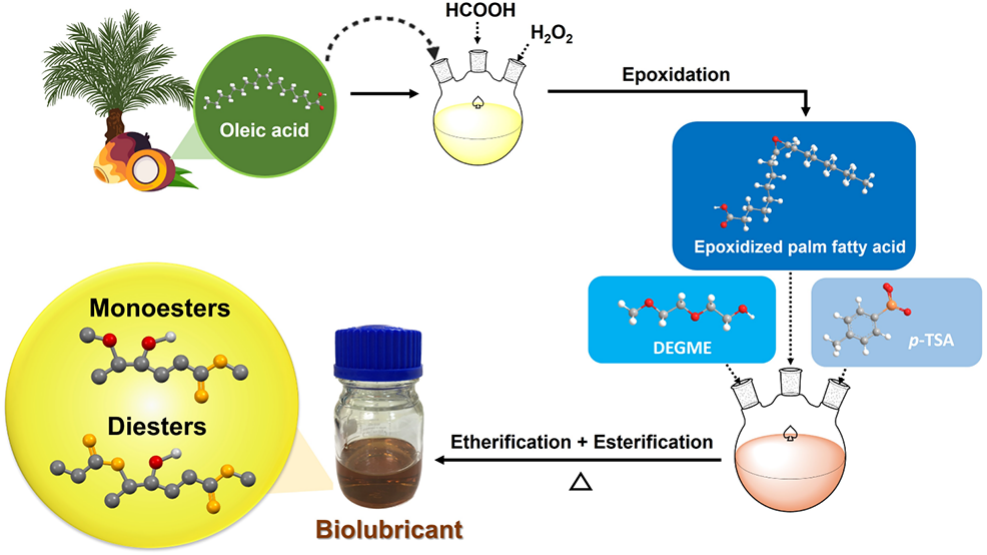

In this work, a new way of biolubricant synthesis is described using diethylene glycol monomethyl ether (DEGME) as a novel coreactant for the dual etherification and esterification of epoxidized mixed fatty acid (EO), catalyzed by *p*-toluenesulfonic acid (*p*-TSA) as a catalyst. A constant EO:DEGME molar ratio of 1:2 was used for the reaction, and different catalyst loadings (2.6 wt %), temperatures (120 °C), and reaction times (5 h) were used. Under the optimum operating condition, a conversion of 100% was reached, and the ester-based biolubricants with a yield of 89.9% were attained. The final lubricants were primarily made of mono- and diesters. Physicochemical characteristics, including kinematic viscosity, viscosity index, pour point, acid value, and moisture content, were assessed using accepted ASTM methods. Among the basic stocks created, ester-based lubricants showed good characteristics such as the kinematic viscosity of 47.8 cSt at 40 °C and 8.3 cSt at 100 °C, a high viscosity index of 150, a pour point of −1 °C, a low acid value of 0.24 ± 0.02 mg KOH/g, and a low moisture content of 0.01 ± 0.005%. Our research provides a cost-effective and environmentally friendly method for creating high-performance biolubricants with tunable kinematic viscosity range, high viscosity index, and low water impurity. This innovative palm fatty acid-based biolubricant leads to potential industrial utilizations such as hydraulic oil, transformer oil, and metal cutting fluid.

## 1 Introduction

The current increased demand for energy, the rising cost of crude oil, greenhouse gas emissions causing global warming, environmental pollution, and the rapidly declining availability of fossil fuels are the main issues driving the quest for alternative energy sources.^1−3^ Finding a sustainable, renewable, and commercially viable alternative energy source is a great way to address this challenge. There are many different forms of renewable energy sources, including hydropower, geothermal, wind, solar, and biomass. Among these, biomass is the only renewable source from which energy and chemical goods may be produced; as a result, it is the only option now available that can replace petroleum in the synthesis of a variety of important organic products.

Lubricants with superior friction reduction and lubrication efficacy are essential to the economic and industrial growth of the nation. The production of durable, high-quality lubricants reflects the nation’s manufacturing and economic status. In their pursuit of environmentally safe, nontoxic lubricants, and renewability, researchers are becoming increasingly interested in biolubricants (lubricants of biological origin) as alternatives to conventional lubricants. Biolubricants are produced from renewable natural feedstocks, such as vegetable oils and animal lipids, and used cooking oil. These biolubricants have high viscosity indexes and exceptional lubricity.^4−9^ The uses of biobased and environmentally friendly biodegradable lubricants have gained attention to prevent soil, river, and ocean pollution from contamination of lubricant wastes and spills from transportation and industrial sectors.^10,11^

Palm oil has been effectively implemented as a renewable resource for the production of biolubricant base stocks of comparable quality to those derived from petroleum.^12,13^ It is widely acknowledged that palm oil is a highly productive commodity, producing a high yield at a lower price than those of other vegetable oils. Crude palm oil (CPO) is the extracted oil from palm oil, and it comprises between 3 and 7 wt % of palm fatty acid distillate (PFAD).^14^ Palmitic and oleic acids are the primary fatty acids. Utilizing PFAD as a feedstock as opposed to vegetable oils has waste refining and sustainability benefits. PFAD is a suitable feedstock for the synthesis of biolubricant base stocks because it produces a robust lubricant layer and acts as a boundary lubricant, interacting directly with contact surfaces. However, their low oxidation resistance and thermal stability, as well as their limited viscosity range, restrict their use as industrial lubricants. Therefore, many scientific methodologies are being developed to produce industrially appropriate PFAD-based lubricants.

To produce biolubricant base stocks from PFAD, several chemical reactions, including polyol ester synthesis via esterification and epoxidized oil conversion, were carried out in a homogeneous or heterogeneous phase using acid catalysts.^15−21^ All reactions involved in the chemical modification of fatty acid molecules are indispensable and impact the characteristics of the basestock. Significant esterification reactions lower the acidity of fatty acid derivatives to make intermediates that can be used in a wide range of businesses.^22^ A biolubricant base stock comprised predominantly of ester compounds improves the thermal stability of the lubricant and meets the minimum requirements for a high-quality lubricant. Petroleum-based polyols like neopentyl glycol (NPG), trimethylol propane (TMP), and pentaerythritol (PE) are often used in the esterification and transesterification of fatty acid methyl esters (FAMEs) to make polyol ester-type biolubricants.^23−27^

Epoxidation reactions have been widely used in the production of ester-based lubricants due to the high reactivity of the epoxy ring.^28^ Etherification is a reaction that produces ether compounds from biolubricant base materials, resulting in enhanced lubricant properties.^29,30^ Since the chemical stability of an ether bond to an alkali is superior to that of an ester bond,^31^ numerous research groups reported that etherification of epoxidized PFAD using alcohols in acid catalysts resulted in chemically modified PFAD with enhanced properties. We have recently reported a method for synthesizing ester-based biolubricants from palm fatty acids by combining HBF_4_-catalyzed oxirane ring-opening with esterification of epoxidized oleic acid and 2-ethyl-1-hexanol.^32^ The biolubricants based on esters attained outstanding viscosities and pour points, among other favorable physicochemical properties. To the best of our knowledge, the use of ether compounds to produce biolubricants via dual etherification and esterification has not yet been reported.

Diethylene glycol monomethyl ether (DEGME) is an odorless, colorless, hygroscopic liquid used as a solvent in textile dye pastes, synthetic resin surface coatings, and lacquers.^33^ Furthermore, DEGME is widely used as a fuel system icing inhibitor (FSII) in military and commercial aircraft.^34,35^ The secondary effect of inhibiting microbial growth is observed.^36^ Consequently, the objective of this study is to produce biolubricant base stock through the dual etherification and esterification of epoxidized palm fatty acid. The novelty of the presented work is that an ether compound such as DEGME containing an alcohol group is employed during the etherification stage by using *p*-toluenesulfonic acid (*p*-TSA) as a catalyst. The effects of operational parameters on the second etherification reaction were examined, and optimal conditions were reported. The physicochemical properties of the biolubricant base stock were then evaluated using standard ASTM methods.

## 2 Experimental Section

### 2.1 Chemicals and Materials

All reagents are of analytical quality and are utilized without additional purification. A technical grade oleic acid containing 90% oleic acid and 10% stearic acid was purchased from Sigma-Aldrich. Formic acid (99.5%, HCOOH), hydrogen peroxide solution (30%, H_2_O_2_), toluene (99.9%), and anhydrous sodium carbonate (99.5%, Na_2_CO_3_) were obtained from Merck. Diethylene glycol monomethyl ether (99%, DEGME) was purchased from the Tokyo Chemical Industry. *p*-toluenesulfonic acid monohydrate (99%, *p*-TSA) was obtained from Acros Organics.

### 2.2 Epoxidation

The epoxidation operations were carried out using a modified version of a previously published approach.^37^ Briefly, 30 g of oleic acid was weighed and mixed into a three-neck round-bottom flask (250 mL) fitted with a thermometer, a magnetic bar, and a reflux condenser. A silicone oil bath and a magnetic hot plate-stirrer were used as a heat source and for stirring. The oleic acid was acidified with formic acid while being continually stirred. H_2_O_2_ was then slowly added at room temperature. The chosen mole ratio of double bonds of the oils (C=C): HCOOH: H_2_O_2_ was 1:2:3. After addition of all of the hydrogen peroxide, the reaction was kept at 45 °C for 3.5 h with vigorous stirring. The mixture was then washed with a warm solution of 5% NaHCO_3_ solution and deionized water to neutralize any remaining acid and remove the organic layer from the mixture. The neutralized organic phase mixture was then purified for 30 min under vacuum using a rotary evaporator at 60 °C to remove traces of water. The conversion and yield of epoxidized oleic acid are 100% and 97−99%, respectively.

### 2.3 Etherification and Esterification

The etherification reaction was performed in a three-necked round-bottom flask and similar equipment setup as previously described in Section 2.2. Diethylene glycol monomethyl ether (DEGME) was added to 30 g of epoxidized oleic acid (EO) obtained from Section 2.2 (mol ratio of DEGME:EO = 2:1). 0.78 g portion (2.6%w/w of EO) of *p*-TSA and 1 g of toluene were added to the mixture. The etherification was performed in a reflux system at 120 °C, 500 rpm, for 5 h. After the reaction was completed, the mixture was cooled and rinsed with a 5 wt % NaHCO_3_ solution and deionized water to eliminate remaining acids (e.g., *p*-TSA) in the organic phase. Finally, rotary evaporation was used to remove trace water from the crude oil product.

### 2.4 Analysis of Biolubricant Base Stock Products

The biolubricant base stock products were examined and quantified using a capillary column (Agilent DB-5HT, 30 m, 0.32 mm ID, 0.1 m DF) and gas chromatography fitted with a mass spectrometer (Shimadzu GCMS-QP2020 NX). A 10 μL portion of the samples was injected into the GC-MS instrument with a split ratio of 50:1. The column temperature was originally set at 40 °C, maintained for 4 min, and then increased at a rate of 20 °C/min to 150 °C. Finally, the temperature was increased by 5 °C/min to 320 °C/min and maintained for 5 min. The injector and detector were both at 250 °C. Helium was employed as the carrier gas at a pressure of 25 kPa.

The conversion and yield of all products were calculated using the following equations:
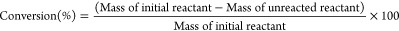
1
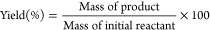
2

Nuclear magnetic resonance (NMR) spectroscopy was used to characterize the samples’ structures. Generally, 20 mg of sample was dissolved in deuterated chloroform (CDCl_3_, 99.8% D, Cambridge Isotope Laboratories). The ^1^H and ^13^C NMR spectra were obtained at 500 MHz on a Bruker Avance DRX-500. Fourier transform infrared (FT-IR) spectra were measured using an ATR mode Bruker FT-50. The spectrum was collected in the 400 to 4000 cm^−1^ wavenumber band for 32 repeated scans at 4 cm^−1^ spectral resolution. Thermal gravimetric analysis (TGA) of the samples was performed using a Metter Toledo—TGA/SDTA. A 2.5 mg sample was utilized in an alumina crucible at a heating rate of 10 °C/min, with a heating range of 30−450 °C under a nitrogen flow rate of 30 mL/min. Differential scanning calorimetry (DSC) studies were evaluated on a DSC 214 Polyma (NETZSCH). A 2 μL sample was sealed in an aluminum pan with a pinhole cover and oxidized in the air atmosphere. In each experiment, a heating rate of 20 °C/min was utilized from 20 to 300 °C. A heat flow (V/mg) versus temperature (°C) plot was used to calculate the oxidation onset (OT, °C) and signal maximum temperatures (SMT, °C).

### 2.5 Biolubricant Base Stock Property Measurements

Physicochemical properties of all samples were carried out using various characterization techniques. A Stabinger Viscometer (SVM 3001, Anton Paar) was used to test the kinematic viscosity (KV) at 40 and 100 °C in accordance with ASTM D7042. The viscosity index (VI) was derived from the KV data at 100 and 40 °C using ASTM D2270. The density was determined using an ASTM D4052 digital density meter (DMA 501, Anton Paar) at 15 °C. The pour point was determined using an Automated Mini Pour/Cloud point tester, TANAKA’s MPC-102S model, in accordance with ASTM D6749, which yields the pour point in a manner comparable to ASTM D97. The moisture content of the samples was determined using an ASTM D4928 standard protocol and a Karl Fisher Titration (890 KF Titrando, Metrohm).

## 3 Results and Discussion

### 3.1 Reaction Pathway

**Scheme 1:**
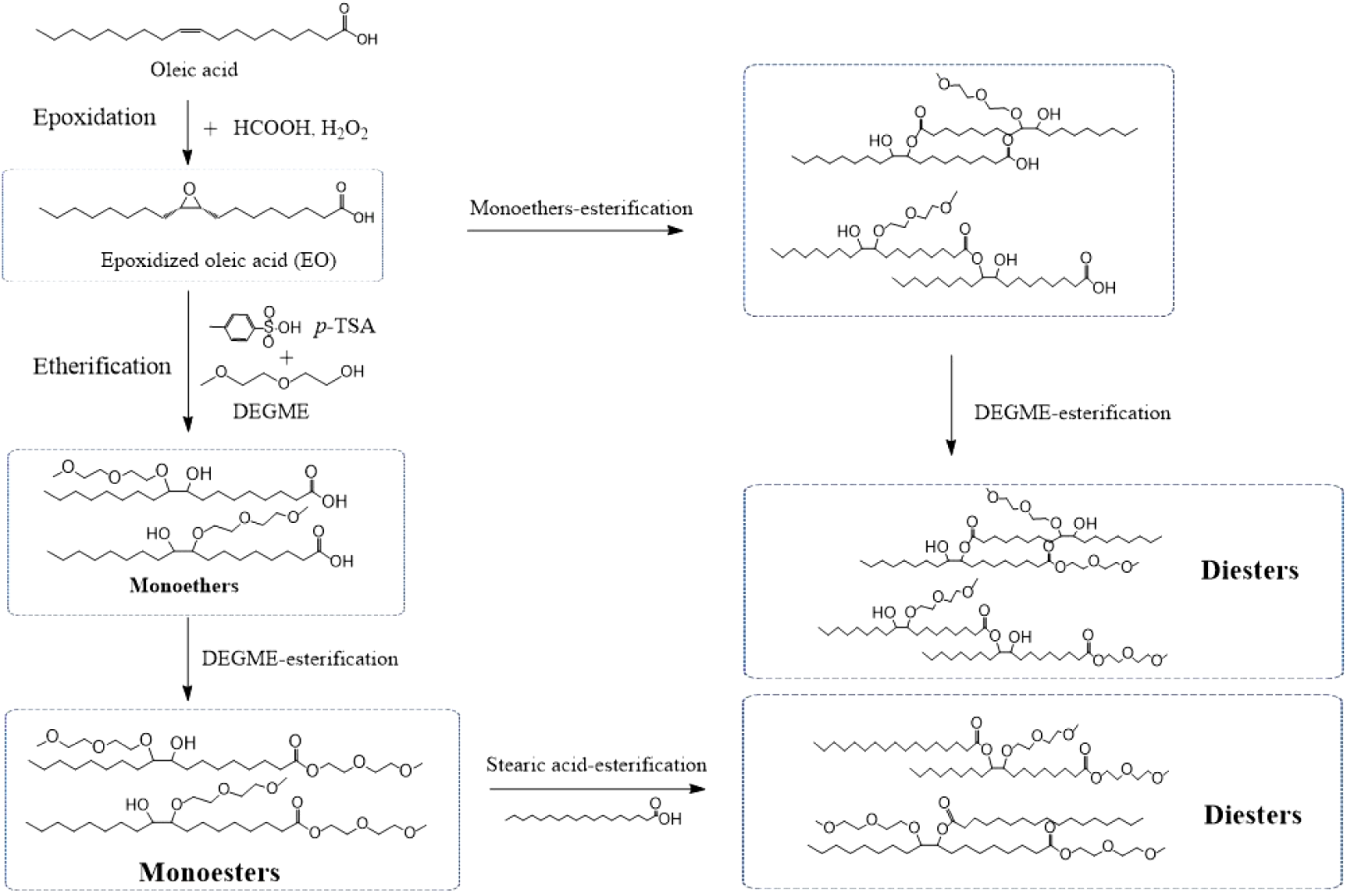
Proposed Reaction Pathway for Producing Biolubricant Base Stocks by Epoxidation and Dual Etherification-Esterification

In this process, technical-grade oleic acid with a 90% oleic acid content and a 10% stearic acid content was used to produce biolubricant base stocks via a two-step procedure. Performic acid (HCOOOH) was initially used to catalyze the epoxidation of oleic acid (EO). To produce HCOOOH, we combine formic acid (HCOOH), which functions as an oxygen transporter, with hydrogen peroxide (H_2_O_2_), which functions as an oxygen donor.^38^ Epoxidized oleic acid (EO) was produced by converting an unsaturated oleic acid link to an oxirane ring. The mechanism occurred as a result of an electrophilic attack that generated carboxylic acid as a byproduct. After breaking the O−O bond, a carbonyl bond is formed. The electrons from the previous O−H bond generated the second new C−O bond, while the proton was absorbed by the original carbonyl group’s electrons. While the transition state of the reaction made the processes of bond formation and bond breaking much clearer, the process of bond formation was facilitated by the state.^39^ In the subsequent step, the biolubricant base stock products were obtained through dual etherification and esterification with DEGME and *p*-TSA as the catalysts. In the initial reaction, the acid (*p*-TSA) interacted with the EO to generate a protonated EO. Then, OH of DEGME acted as a nucleophile, opening the ring and generating hydroxyl and ether groups. The terminal carboxyl group (−COOH) of EO simultaneously reacted with the alcohol of DEGME through an esterification process, releasing water and forming an ester compound as a monoester product (Monoesters). In addition, the hydroxyl group of monoesters reacted with COOH of the terminal carboxyl group of EO or residual stearic acid to produce Diester A or Diester B via an esterification process. The ketonization of epoxidized oleic acid during the production of biolubricant stock resulted in the formation of ketone ester under unfavorable conditions, such as a reduced catalyst amount and a lower temperature. The proposed reaction pathway for producing biolubricant base stock is depicted in Scheme 1, and all biolubricant base stock products are listed in Table S1.

### 3.2 Catalytic Performance of Biolubricant Base Stock Production

#### 3.2.1 Effect of the Catalyst Amount

**Figure 1:**
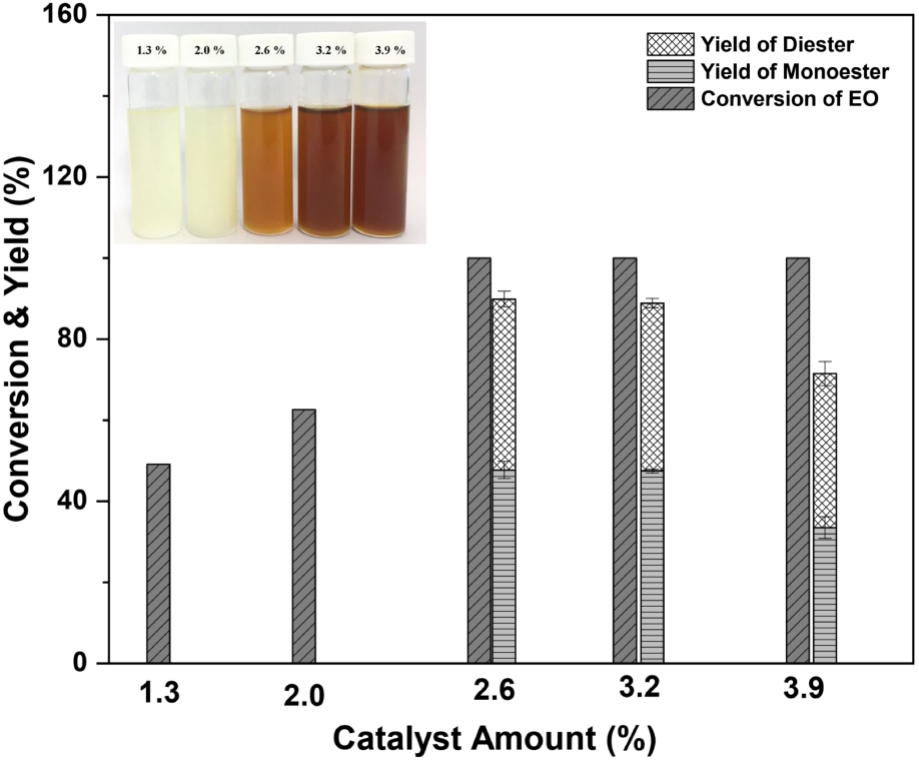
Effects of catalyst amount on yield of biolubricant base stock products (BL1:1.3 wt %, BL2:2.0 wt %, BL3:2.6 wt %, BL4:3.2 wt %, and BL5:3.9 wt %). Reaction condition: molar ratio of EO/DEGME of 1:2 at 120 °C for 5 h.

At an EO/DEGME molar ratio of 1:2 at 120 °C for 5 h, the effects of the amount of catalyst (*p*-TSA) on the reaction were studied. As seen in Figure 1, a larger amount of catalyst improves oxirane conversion as a result of the increased availability of catalytic active sites. When applying a catalyst loading of less than 2.6 wt %, a minimal conversion of EO (90%) without any desirable product was observed (Table 1, BL1 and BL2). When the amount of catalyst reached 2.6 wt % of the EO, the yield of the total oil product (monoesters and diesters) reached 89.9% with a 100% EO conversion (Table 1, BL3), as the number of acid sites in the process was sufficient to convert EO to desirable products, resulting in increased product yields. In addition, as the quantity of catalyst increased from 2.6% (BL4) to 3.9% (BL5), the biolubricant yield decreased dramatically (Figure 1). This was due to the high acidity, which could induce side effects. Intriguingly, as the catalyst dosage increased, the biolubricant’s color changed from pale yellow to brownish-black, indirectly reflecting the catalytic performance. Consequently, a catalyst concentration of 2.6% EO was selected for further testing.

#### 3.2.2 Effect of the Reaction Temperature

**Figure 2:**
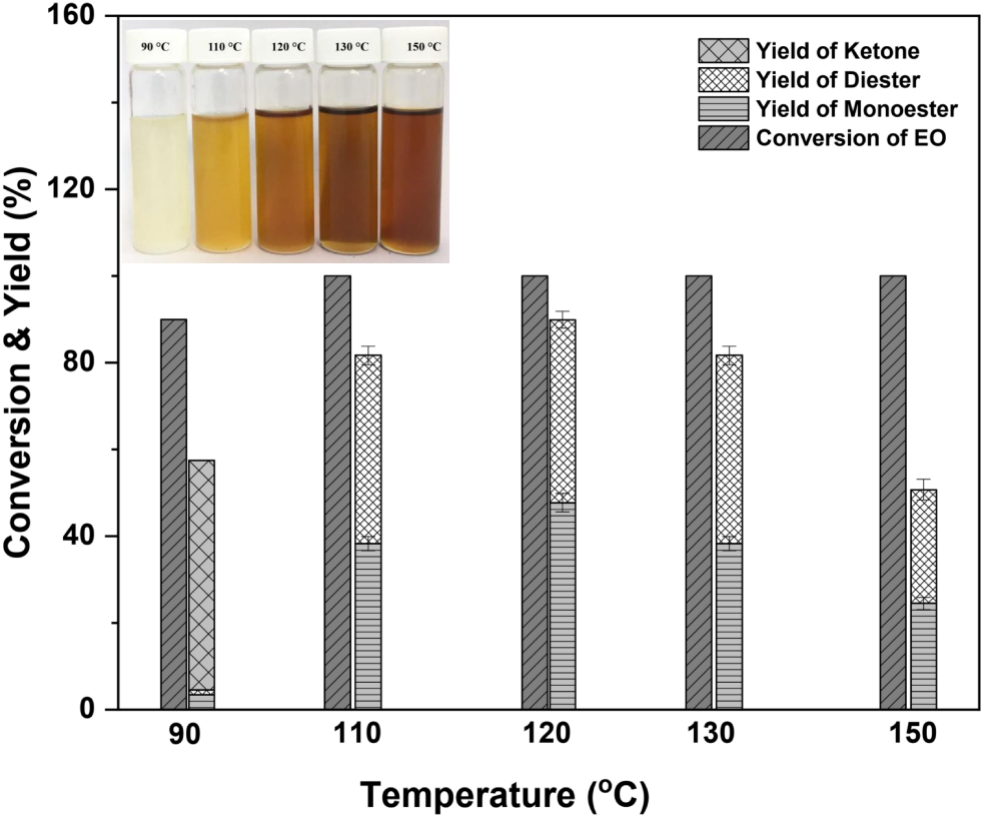
Effect of reaction temperature on biolubricant base stock product yield (BL6:90 °C, BL7:110 °C, BL3:120 °C, BL8:130 °C, and BL9:150 °C). Reaction conditions: molar ratio of EO/DEGME of 1:2, 2.6 wt % of *p*-TSA, and a reaction time of 5 h.

The effects of reaction temperature were investigated between 90 and 150 °C. Experimental parameters included an EO/DEGME molar ratio of 1:2, a *p*-TSA catalyst dose of 2.6% of the EO mass, and 5 h reaction time. Figure 2 demonstrates that increasing the reaction temperature from 90 to 120 °C increased product yields from 4.5% to 89.9.8%. At a low temperature of 90 °C, the reaction produced ester compounds with a yield of 4.5% and an EO conversion of 90% (BL6 in Table 1). During the second stage, epoxide ring rearrangement led to the formation of ketone as a significant byproduct. When the reaction temperature was 110 °C (BL7), the monoester and diester yields were 84.3%. At 120 °C, the monoester and diester products were at their peaks (89.9%, BL3); at 130 °C, they decreased slightly (85.0%, BL8). In addition, increasing the temperature to 150 °C decreased the yields of the principal products (monoesters and diesters) by 48% (BL9) due to side reactions such as the formation of diether compounds (Table S1). At the reaction temperatures (110−150 °C), there was no detection of the ketone product. In addition, the biolubricant base stock products darkened in color. Therefore, 120 °C was used as the reaction temperature in subsequent investigations.

#### 3.2.3 Effect of the Reaction Time

**Figure 3:**
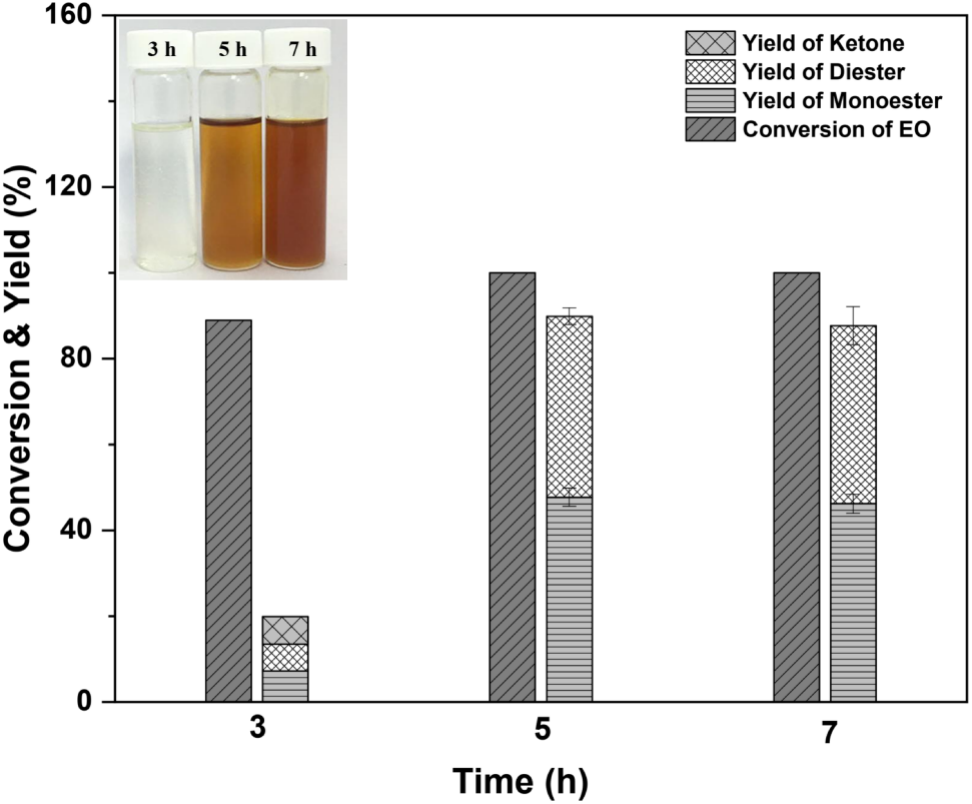
Effect of reaction time on biolubricant base stock product yield (BL10:3 h, BL7:5 h, and BL11:7 h). Reaction conditions: molar ratio of EO/DEGME of 1:2, 2.6 wt % of *p*-TSA at 110 °C.

The effect of reaction time was investigated using an EO/DEGME molar ratio of 1:2, a *p*-TSA catalyst amount of 2.6% wt. of the EO mass, a reaction time range of 3−7 h, and a reaction temperature of 110 °C. As shown in Figure 3, as the reaction duration increased from 3 to 7 h, the EO conversion and product yields increased significantly. The product transformed from pale yellow to dark brown in color. Ester products were produced with a yield of 13.5% and an EO conversion of 89.0% (BL10 in Table 1) after 3 h of reaction time. According to reports, a trace quantity of the ketone product was also detected. Product yields rose to 92.7% after 5 h of reaction time and remained constant after 7 h (92.5%, BL11) after 5 h of reaction time. To maximize energy savings and biolubricant base stock production, the optimal reaction time of 5 h was chosen.

**Table 1:** Conversion and Yield of Biolubricant Base Stock Products from Etherification Combined with Esterification Reaction with DEGME at Various Reaction Conditions^a^

			Product yields		
Samples	Conditions	Conversion (%)	Monoester	Diester	Ketone
BL1	120 °C, 5 h, 1.3 wt %	49.1	ND	ND	ND
BL2	120 °C, 5 h, 2.0 wt %	62.6	ND	ND	ND
BL3	120 °C, 5 h, 2.6 wt %	100	47.7 ± 2.12	42.2 ± 1.97	ND
BL4	120 °C, 5 h, 3.2 wt %	100	47.5 ± 0.49	41.4 ± 1.13	ND
BL5	120 °C, 5 h, 3.9 wt %	100	33.5 ± 2.68	38.0 ± 2.97	ND
BL6	90 °C, 5 h, 2.6 wt %	90	3.4	1.1	53
BL7	110 °C, 5 h, 2.6 wt %	100	38.3 ± 1.63	43.4 ± 2.12	ND
BL8	130 °C, 5 h, 2.6 wt %	100	44.75 ± 3.32	39.3 ± 1.91	ND
BL9	150 °C, 5 h, 2.6 wt %	100	24.5 ± 1.42	26.2 ± 2.40	ND
BL10	110 °C, 3 h, 2.6 wt %	89	7.2	6.3	6.4
BL11	110 °C, 7 h, 2.6 wt %	100	46.2 ± 2.26	41.5 ± 4.52	ND

a ND = not detected.

### 3.3 Structure Characterization

Biolubricant base stocks were synthesized through dual etherification and esterification of epoxidized oleic acid (Scheme 1). The conversion and yield of epoxidized oleic acid are 100% and 97−99%, respectively. The etherification reaction was proceeded by direct titration of epoxy and terminated when the reaction reached 100%. The structure chemistry of the product was analyzed by the GC-MS chromatogram as shown in Figures S1 and S2.

**Figure 4:**
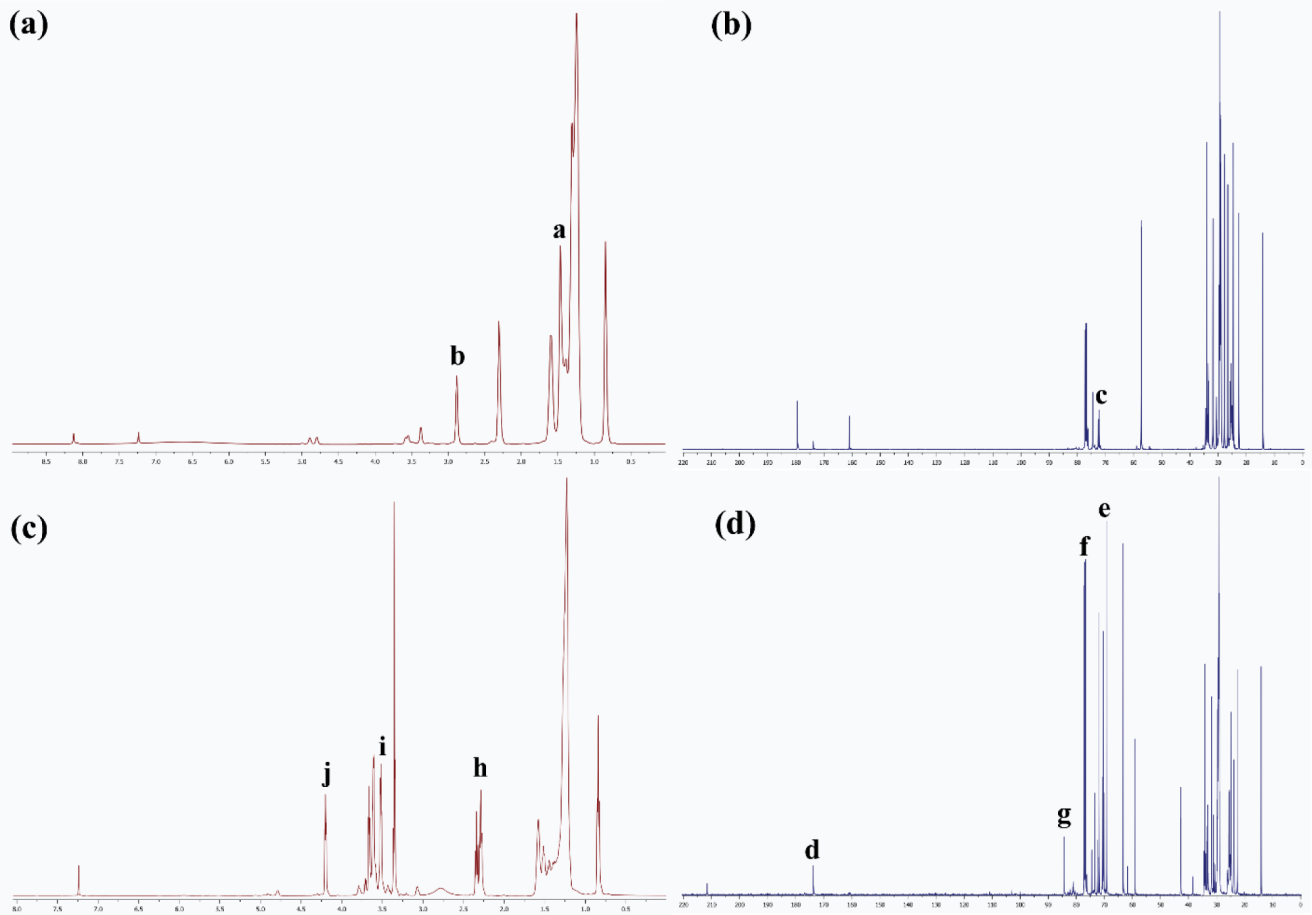
NMR spectra of epoxidized oleic acid: (a) ^1^H NMR spectrum and (b) ^13^C NMR spectrum, and biolubricant base stock: (c) ^1^H NMR spectrum and (d) ^13^C NMR spectrum.

The formation of the EO and biolubricant base stock was evaluated by ^1^H NMR and ^13^C NMR. The ^1^H NMR spectrum of EO (Figure 4a) showed the protons nearby an epoxy group as represented in the chemical shift of 1.4−1.5 ppm (region **a**, −C**H**_**2**_−CHOCH−C**H**_**2**_) and the chemical shift ∼2.9 ppm (region **b**) corresponded to a proton attached to the oxirane-carbons (−C**H**OC**H**−).^40^ Those peaks in region **a** and region **b** were absent in the ^1^H NMR spectrum of BL3 (Figure 4c). Multiple peaks at ∼2.3−2.4 ppm (region **h**, −C**H**(=O)O−C**H**−), ∼3.4−3.6 ppm (region **i**, −O−C**H**_**2**_−), and ∼4.4 ppm (region **j**, −OC(=O)−C**H**_**2**_−) indicated the formation of new ester and ether biolubricant molecules due to ring-opening by DEGME. The ^13^C NMR spectrum of EO (Figure 4b) represents the peaks of carbons at the epoxide ring (−**C**HO**C**H−) around 76−77 ppm (region **c**,) as a result of the epoxidation procedure converting the oleic acid double bond to epoxide groups and there is no peak around 129−130 ppm of typical double bond carbon (−C=C−) as observed in the oleic acid (Figure 4c). After the etherification and esterification, the ^13^C NMR spectrum of BL3 (Figure 4d) reveals the formation of ester carbons (−**C**OO−) around 174 ppm (region **d**) and ether carbons (−**C**−O−**C**−) of the DEGME fraction around 69−74 ppm (region **e**) and ether carbons resulted from etherification (−**C**(O**C**−R)−C(OH)−C−, region **f**) at 77−78 ppm. Moreover, the ring-opened carbon at the center of biolubricant molecules was confirmed by the observed characteristic peak hydroxy carbon (−C(OR)−**C**(OH)−C−, region **g**) at ∼85 ppm.

**Figure 5:**
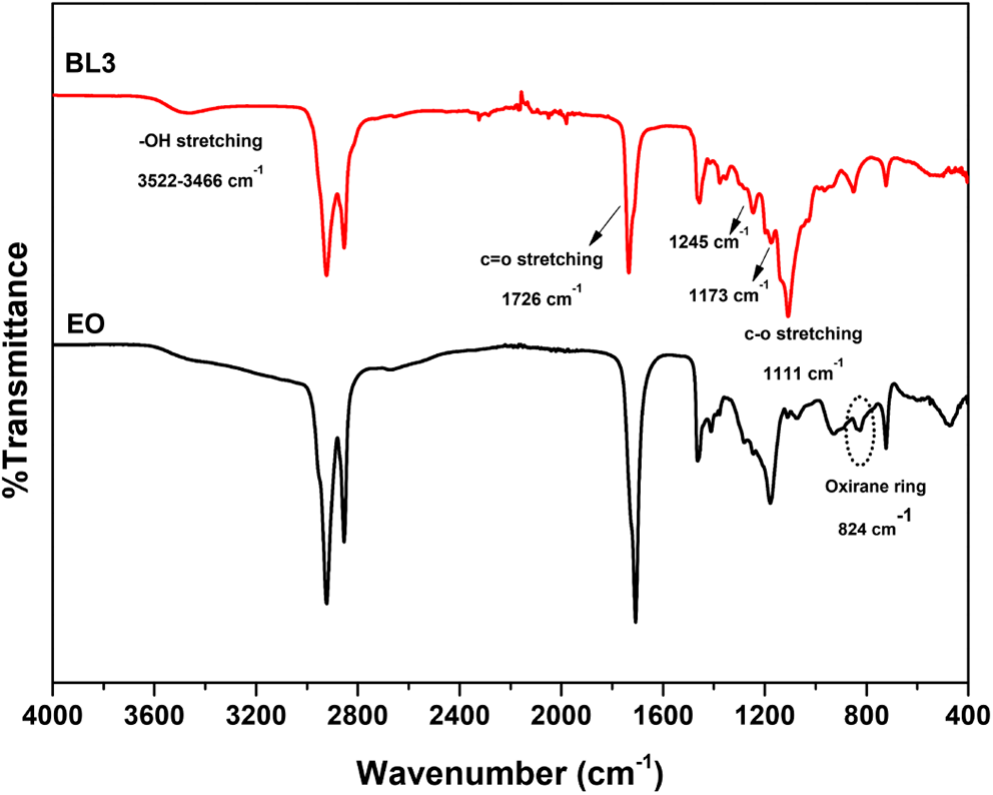
FT-IR spectra of epoxidized oleic acid and biolubricant base stock (BL3).

Figure 5 shows the FTIR spectra of EO and the products of etherification and esterification with DEGME. The EO spectra have a distinctive band at ∼824 cm^−1^, which is ascribed to the quaternary carbons of the epoxy stretching.^41^ Theoretical explanations suggest that the epoxy could be observed at wavenumbers of 750−880 cm^−1^ and 815−950 cm^−1^.^42^ The FTIR spectrum of the biolubricant base stock showed a vibration peak of C=O at ∼1726 cm^−1^, a stretching vibration peak of C−O for ether at 1111 cm^−1^ and 1173 cm^−1^, a stretching vibration peak of C−O for ester at 1245 cm^−1^, and −OH stretching of alcohol at ∼3466−3522 cm^−1^.

### 3.4 Physicochemical Properties of As-Synthesized Biolubricant Base Stock

The lubricant properties of the biolubricant base stock products are shown in Table 2. When product yields varied between 89.4% and 92.8%, the kinematic viscosity temperature at 40 and 100 °C was 40.7−47.3 and 7.7−8.3, respectively. Variations in the viscosity index were based on conversion and biolubricant content (yields of monoester and diester products). One of the preferable characteristics of lubricants is a viscosity index greater than or equal to 150 for synthetic oil. In general, the observed high viscosity index demonstrated that the viscosity of oil products does not alter abruptly as a result of their molecular structures.^43^ A machine’s performance is consistent and high when its viscosity varies minimally over a broad temperature range. In this study, the high total yields of monoesters and diesters (>85%; BL3, BL4, BL11) led to an extremely high viscosity index of over 150 when DEGME was attached to the molecular structure of EO through esterification and etherification, as well as an appropriate ratio of monoester to diester components in the biolubricant product. These products, with a viscosity index of 150, are suitable for use as hydraulic fluids and metalworking fluids.^44^ In addition, the samples with an outstanding viscosity index (e.g., viscosity index >150) were diether compounds (BL5 and BL9). BL6 contained ketone compounds with unusually high kinematic viscosity (>2200 cSt at 40 °C) and a low viscosity index. A cold flow property, as determined by a pour point, is one of the essential lubricant properties related to the working temperature of oil in low-temperature conditions. Practically all applications, from hydraulic fluids to aviation and turbine lubricants, require extremely low flow points. These low pour points may be the result of microcrystalline structures being perturbed by extensively branched formations.^45^ In this investigation, the pour points of the biolubricant base stocks as synthesized ranged from 15 to −1 °C (Table 2).

**Table 2:** Physicochemical Properties of Biolubricant Base Stock Products^a^

Samples	KV at 40 °C (cSt)	KV at 100 °C (cSt)	VI	PP (°C)	Acid value (mg KOH/g)	Water content (%)
BL1	429.5	24.4	69.3	5	0.42 ± 0.015	0.03 ± 0.010
BL2	249.9	20.1	93.2	4	0.37 ± 0.020	0.01 ± 0.005
BL3	47.3	8.3	150.0	−1	0.25 ± 0.025	0.01 ± 0.005
BL4	40.7	7.5	151.4	1	0.45 ± 0.031	0.04 ± 0.006
BL5	41.0	7.7	151.5	2	0.46 ± 0.019	0.01±0.005
BL6	2212.0	93.9	108.9	7	0.41 ± 0.035	0.05 ± 0.023
BL7	53.0	8.8	144.5	4	0.36 ± 0.017	0.01 ± 0.005
BL8	44.7	7.9	146.5	−1	0.32 ± 0.022	0.03 ± 0.08
BL9	40.6	7.5	152.2	3	0.16 ± 0.017	0.03 ± 0.09
BL10	33.6	5.8	116.9	15	0.33 ± 0.005	0.06 ± 0.005
BL11	41.3	7.5	151.3	3	0.44 ± 0.021	0.02 ± 0.005

a KV = Kinetic viscosity; VI = Viscosity index; PP = Pour point.

### 3.5 Thermal Oxidative Stability

**Figure 6:**
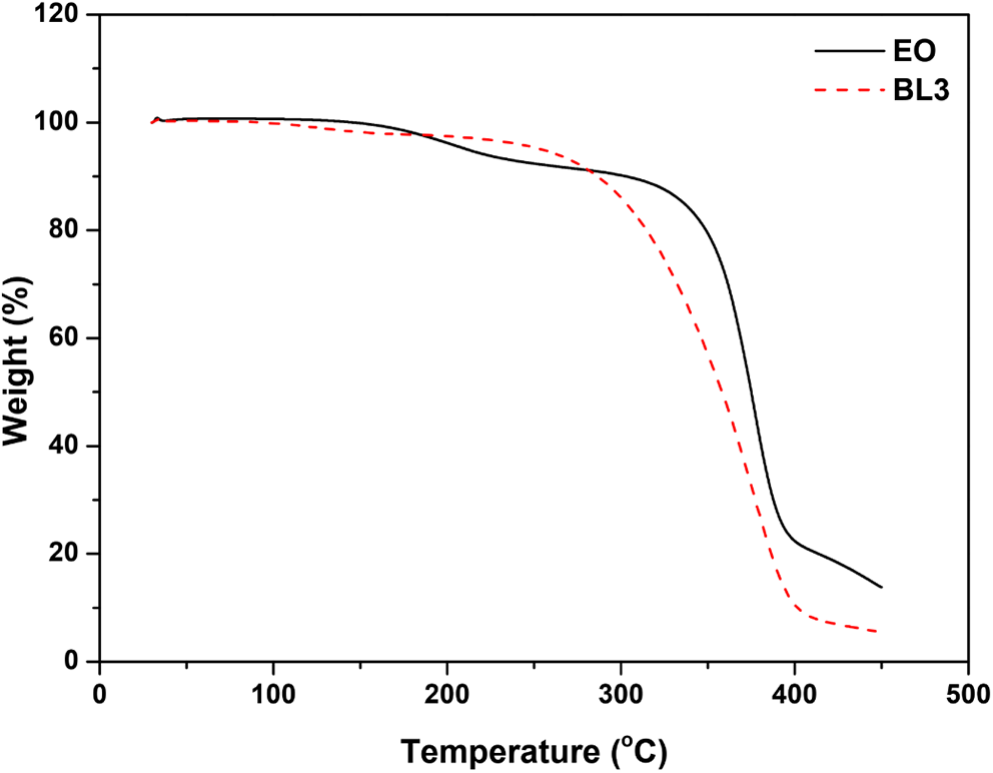
TGA profiles of epoxidized oleic acid and biolubricant base stock (BL3) at 30 mL/min nitrogen flow, 10 °C/min heating rate, and 30−450 °C heating range.

**Figure 7:**
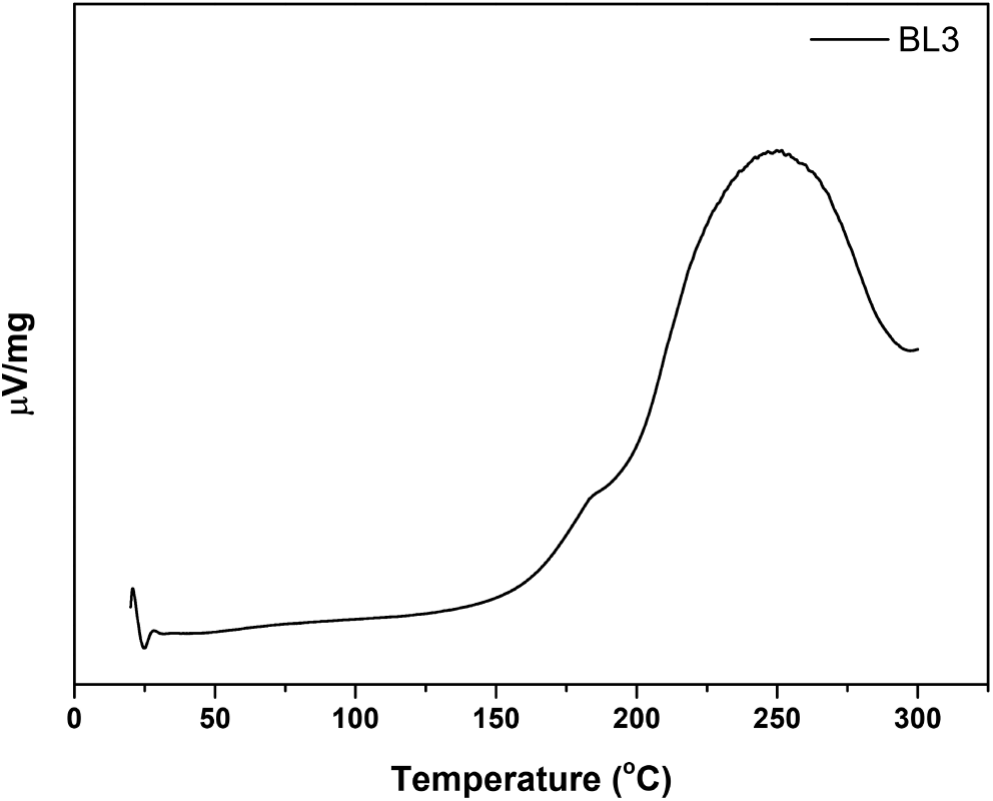
DSC profile of the biolubricant base stock (BL3) under 40 mL/min air flow, 20 °C/min heating rate, and 20−300 °C heating range.

Using TGA, the thermal behavior of the biolubricant base stock was investigated. At high temperatures, the rupturing and/or formation of various physical and chemical bonds results in the evolution of volatile chemicals or the formation of heavier reaction products.^46^ According to the temperature difference between the sample and the reference,^47^ the sample would either emit or absorb energy. Using the OT of thermal degradation in a nitrogen flow, we evaluated the thermal stability of EO, and biolubricant base stocks were evaluated. Figure 6 TGA profiles reveal that EO and BL3 were thermostable at 340 and 260 °C, respectively. However, samples continued to decompose after the onset of temperature. EO was discovered to be more thermally stable than BL3. The increased thermal stability of EO can be attributed to a decrease in the proportion of unsaturated fatty acids in its fatty acid chain, which increases the thermal stability of epoxide.^48^Figure S3 shows the thermograms of all of the synthesized biolubricants. Another crucial characteristic of biolubricants is their resistance to oxidative degradation. Therefore, the oxidation stability of biolubricant base stock (BL3) was determined using DSC by measuring the oxidation onset temperature (OT) and signal maximum temperature (SMT). The OT is the temperature at which the oxidation rate significantly increases. A high OT indicates that a material is resistant to oxidation. The SMT is the temperature at which the sample generates the greatest amount of heat during oxidative decomposition. The OT and SMT of biolubricant base stock (BL3) were observed at temperatures of 168.0 and 251.6 °C, respectively (Figure 7). The obtained OT and SMT showed that the oxidative stability of BL3 was reasonable and could be explained by the oxygenated functional groups of the oil molecules.

Also, all of the samples had acid values between 0.16 ± 0.017 mg KOH/g and 0.46 ± 0.019 mg KOH/g and water contents between 0.01 ± 0.005% and 0.06 ± 0.005%, which met the general standard for both conventional and biobased lubricant base stocks. The biolubricants created by this research could serve as a base stock for hydraulic fluid applications and as lubricants for agricultural machinery due to their physical characteristics, oil composition, and molecular structure. In Table 3, the biolubricant base stock obtained from this work without additives showed excellent and promising physicochemical properties compared with the finished lubricants (made by formulation of base stocks with additives) in several applications. BL3 with a KV (40 °C) of 47.3 cSt could be used as a base stock in a viscosity grade 46 (ISO VG 46, 41.4−50.6 cSt) lubricant in mineral oil-based hydraulic oils. Other potential applications, such as biotransformer oil and metal cutting fluid, could be eligible by improvements in pour point and reduction of KV. These two applications involve a heat transferring function; lowering the viscosity of oil can accelerate heat carrying from one place to another.

**Table 3:** Physicochemical Properties of the Biolubricant Base Stock in This Work in Comparison to Commercial Finished Lubricants on the Market

Properties	BL3 (this work)	PTT Lubricants Hydraulic VG 46	Mobil DTE 25 Ultra VG 46	ABB BIOTEMP	PTT Lubricants HIKUT N11
Application	base oil	hydraulic oil	hydraulic oil	biotransformer oil	metal cutting fluid
KV at 40 °C (cSt)	47.3	46.55	46.2	45	42
KV at 100 °C (cSt)	8.3	7.0	7.1	10	n/a
Viscosity Index	150	108	110	n/a	n/a
Pour point (°C)	−1	−9	−33	−15 to −25	n/a
Water content (%)	0.01 ± 0.005	n/a	n/a	0.015	n/a
Acid value (mgKOH/g)	0.25	n/a	0.7	n/a	n/a
Flash point (°C)	>260 (estimated by TGA)	245 (COC method)	238 (COC method)	330 (COC method)	n/a

## 4 Conclusions

In this study, a novel biolubricant base stock was successfully synthesized by dual etherification and esterification of epoxidized palm fatty acid using DEGME affixed to the molecular structures of EO and *p*-TSA as a catalyst. The results indicated that 5 h of reaction time, 120 °C of reaction temperature, a molar ratio of 1 EO to 2 DEGME, and a catalyst concentration of 2.6% EO were optimal for the etherification and esterification of ester products as biolubricants. The optimized conditions led to a complete conversion of EO and a yield of ester-based biolubricant base stock in excess of 89.9%, with monoester and diester compounds as the primary products. The optimally synthesized BL3 sample exhibited a kinematic viscosity of 47.3 cSt at 40 °C, a viscosity index of 150, a pour point of −1 °C, an acid value of 0.24 mg KOH/g, a water content of 0.01%, and thermal decomposition at 260 °C. Under the optimal reaction conditions outlined in this study, the proposed method can provide a straightforward, inexpensive, biodegradable, and environmentally friendly alternative for the production of biobased lubricants composed of long-chain ethers and esters for lubrication products.

## Supporting Information Available

GC−MS chromatograms of epoxidized oleic acid (EO) and selected biolubricant base stocks, thermogravimetric curves for biolubricant samples prepared at various catalytic conditions, and biolubricant components (PDF)
