# Supplementary material for: Preparation and Physicochemical Properties of a New Biolubricant from Epoxidized Fatty Acids and Diethylene Glycol Monomethyl Ether
Source: ACS Omega. 2025 Apr 29;10(18):19010–8. doi: 10.1021/acsomega.5c01223 (PMC12079590; doi:10.1021/acsomega.5c01223)
Supplement: Supplementary file 1 — ao5c01223_si_001.pdf [file ao5c01223_si_001.pdf]

Electronic Supplementary Information

Preparation and Physicochemical Properties of a New Biolubricant from Epoxidized Fatty Acids and Diethylene Glycol Monomethyl Ether

Kanokwan Chaiendoo, Jeerati Ob-eye, Vorranutch Itthibenchapong*

[a] *National Nanotechnology Center (NANOTEC), National Science and Technology Development Agency (NSTDA), Pathum Thani 12120, Thailand*

*Corresponding author: V. Itthibenchapong

Tel: +66 2 564 7100, Fax: +66 2 564 6981

E-mail: vorranutch@nanotec.or.th

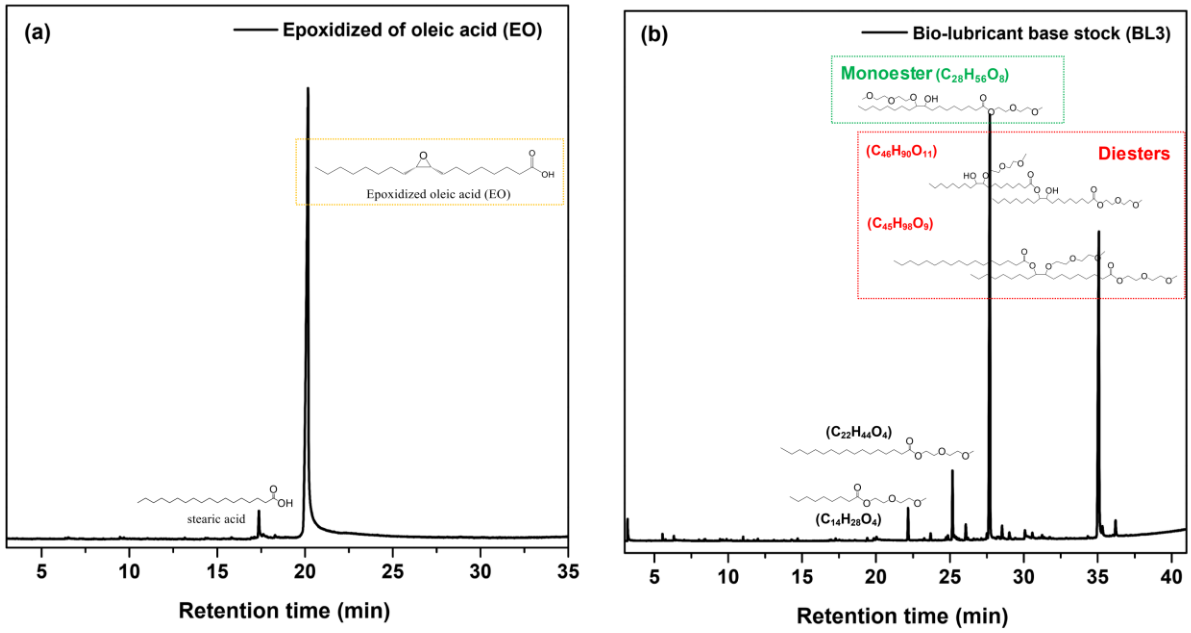

Figure S1 GC–MS chromatograms of epoxidized oleic acid (EO) and bio-lubricant base stock (BL3) via 2 step reactions.

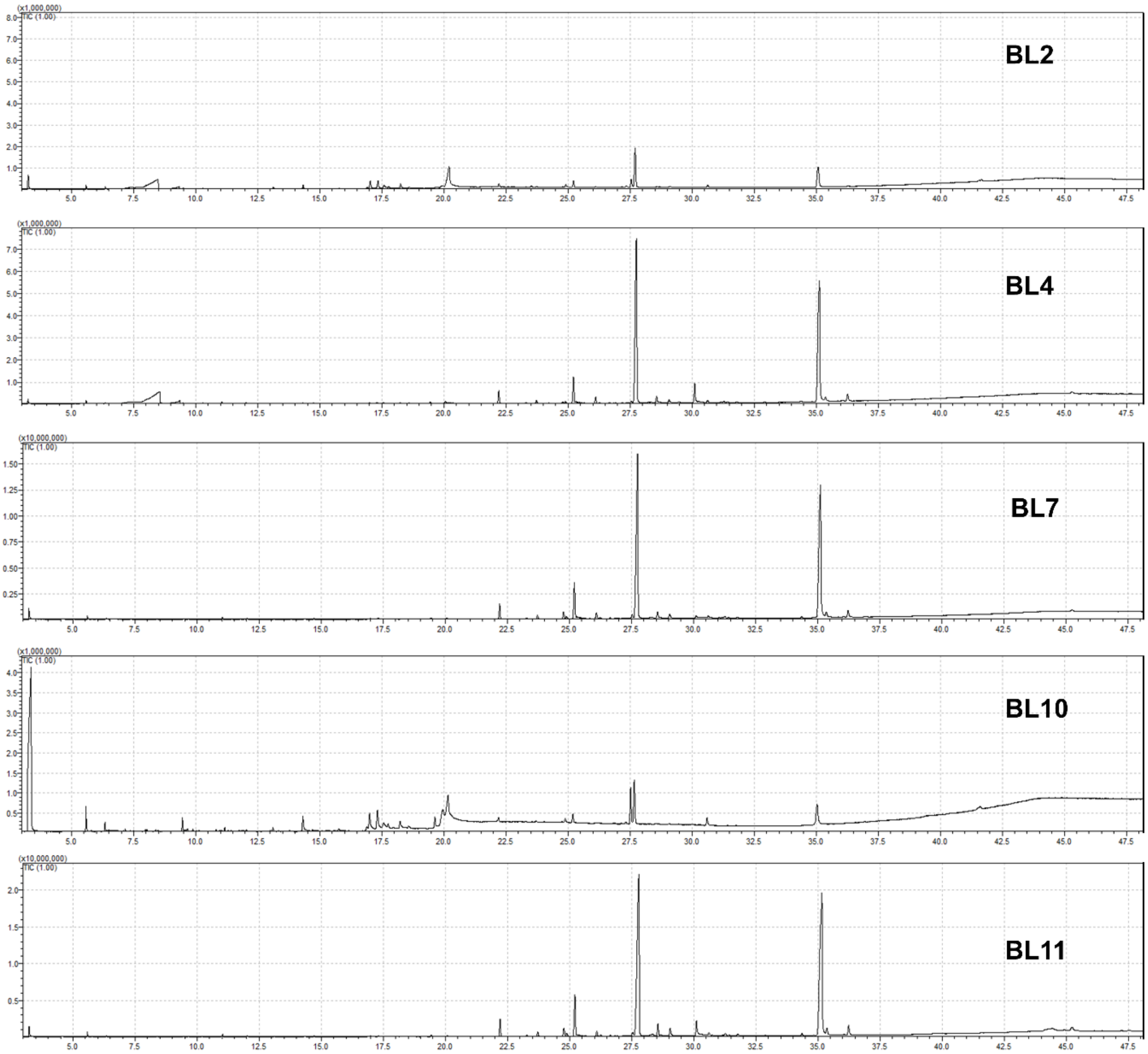

Figure S2 Examples of GC-MS chromatograms of selected bio-lubricant samples.

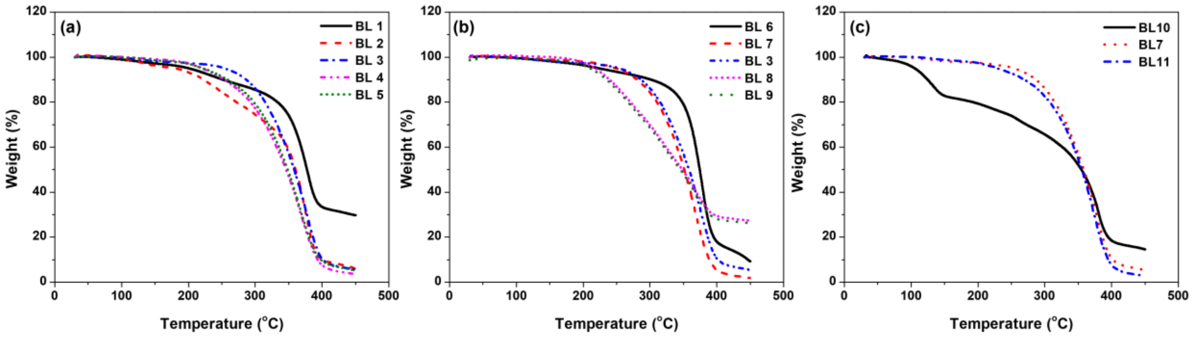

Figure S3 Thermogravimetric curves for bio-lubricant base stock (a) *p*-TSA 1.3-3.9 wt.% (b) T 90-150 °C and (c) Time 3-7 h, under nitrogen flow 30 mL/min with heating rate of 10 °C/min and heating range 30-450 °C.

Table S1. All products of bio-lubricant base stock.

All Products
Monoesters   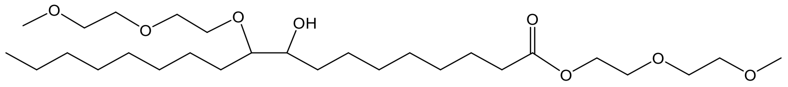   2-(2-methoxyethoxy)ethyl 9-hydroxy-10-(2-(2-methoxyethoxy)ethoxy)octadecenoate ($C_{28}H_{56}O_8$)   or   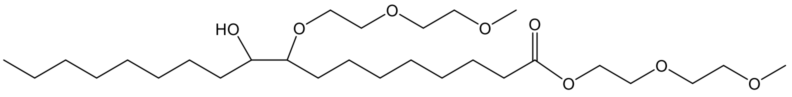   2-(2-methoxyethoxy)ethyl 10-hydroxy-9-(2-(2-methoxyethoxy)ethoxy)octadecenoate ($C_{28}H_{56}O_8$)
Diesters A   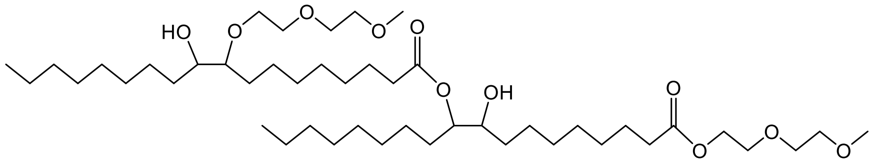   *10-hydroxy-18-(2-(2-methoxyethoxy)ethoxy)-18-oxooctadecan-9-yl 10-hydroxy-9-(2-(2-methoxyethoxy)ethoxy)octadecenoate ($C_{46}H_{90}O_{11}$)*

Or

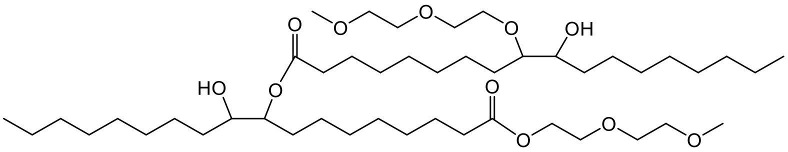

10-hydroxy-1-(2-(2-methoxyethoxy)ethoxy)-1-oxooctadecan-9-yl 10-hydroxy-9-(2-(2-methoxyethoxy)ethoxy)octadecanoate ($C_{46}H_{90}O_{11}$)

Diesters B

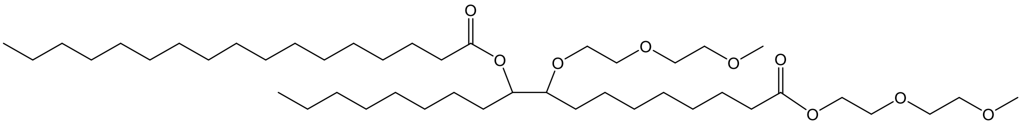

2-(2-methoxyethoxy)ethyl 10-(heptadecanoyloxy)-9-(2(2methoxyethoxy)ethoxy)octadecenoate($C_{45}H_{88}O_{9}$)

or

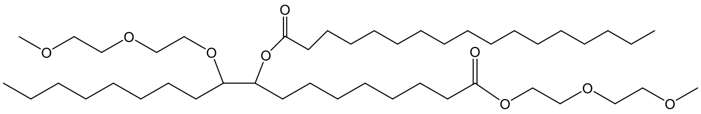

2-(2-methoxyethoxy)ethyl 9-(heptadecanoyloxy)-10-(2-(2-methoxyethoxy)ethoxy)octadecanoate ($C_{45}H_{88}O_{10}$)

Ketone

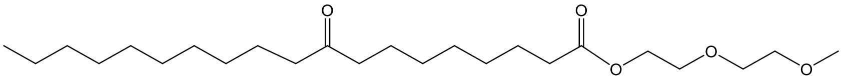

2-(2-methoxyethoxy)ethyl 9-oxononadecanoate ($C_{24}H_{44}O_{5}$)

Diether

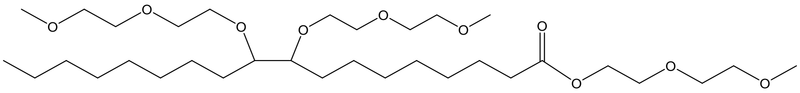

2-(2-methoxyethoxy)ethyl 9,10-bis(2-(2-methoxyethoxy)ethoxy)octadecenoate ($C_{33}H_{66}O_{10}$)
